# Commentary: Reflecting on the Neglected Digital Divide Barriers of Telemedicine During COVID-19

**DOI:** 10.3389/fpubh.2022.915401

**Published:** 2022-05-20

**Authors:** Ali Cheshmehzangi, Tong Zou, Yuxi Zhang, Hengcai Chen, Zhaohui Su, Ayotunde Dawodu, Linjun Xie

**Affiliations:** ^1^Network for Education and Research on Peace and Sustainability (NERPS), Hiroshima University, Hiroshima, Japan; ^2^Department of Architecture and Built Environment, University of Nottingham Ningbo China, Ningbo, China; ^3^Center on Smart and Connected Health Technologies, Mays Cancer Center, School of Nursing, University of Texas Health San Antonio, San Antonio, TX, United States

**Keywords:** telemedicine, telehealth, digital health, digital divide, digital divide barriers, online healthcare

The COVID-19 pandemic has boosted information and communication technologies (ICT) and digital technologies integration in everyday practices. Digital technology-based telemedicine (or telehealth) is one of the main areas that have grown significantly since the start of the pandemic (1–3). Nonetheless, it has widened the digital divide for multiple groups, such as those from remote locations, vulnerable socio-economic background, people with severe physical or mental disabilities, as well as regions with poor access to internet technologies (4, 5). In particular, the elderly groups (or older adults) are affected the most, where we see growing challenges in long-term care facilities (LTCFs) (6), elderly care centers, and private households. Despite the growing attention on the development of ICT infrastructure, availability of ICT devices, and technology interest (7), there are still barriers to the effective use of telemedicine, particularly for vulnerable groups.

In response to the arguments by Shen et al. (3), digital technology-based telemedicine is incorporated in different ways to optimize clinical workflow. The four suggested modes are considered as (1) “many to one” mode, (2) “one to many” mode, (3) “consultation” mode, and (4) “practical operation” mode. In all cases, effectiveness and higher efficiency in data sharing and exchange are evident, but we argue against the point that such platforms are all-inclusive. Hence, this commentary highlights the neglected digital divide in healthcare systems through telemedicine and telehealth platforms, growing much faster than before.

According to Van Dijk and Hacker (8), there are four types of digital divide barriers, including mental (e.g., interests, attractiveness), material (e.g., possession of hardware), skill (e.g., user-friendliness, education, and social support), and usage (e.g., usage opportunities) (9). While we appreciate telemedicine or telehealth as an excellent platform for data exchange and reducing direct interactions between healthcare services/providers/workers and patients, we note that it also creates barriers to multiple groups. Telehealth creates barriers to those with no or little access to digital devices, whether this is related to economic issues, socio-economic issues, or acceptance and use factors. It also becomes a significant burden to those with language barriers, learning difficulties, and digital illiteracy. In particular, such a barrier affects the elderly or older adults as they may be reluctant to use digital/online platforms for healthcare services. They remain a vulnerable group amid the COVID-19 era, who may become more disconnected from their healthcare services. Apart from mental and material digital barriers, skill barriers could be beyond the fundamental skills and more related to the user-friendliness of telehealth platforms. There is often a lack of social support for those in need, leading to further disconnections between the vulnerable groups and the online healthcare services. Such barriers could particularly affect two modes of “many to one mode” and “consultation mode,” where one-to-one online interactions are deemed essential. Lastly, barriers to usage opportunities could be augmented easily due to lack of utilization (including use and utility of the internet), availability, accessibility, and support. Thus, telehealth is not necessarily an all-inclusive digital platform.

Despite the benefits of “*telemedicine in the context of a huge health crisis*” (3), the digital divide barriers caused by telemedicine cannot and must not be neglected. Its effectiveness is mainly related to “data exchange” and “data sharing,” which is indeed efficient and impactful amid the COVID-19 pandemic. However, many people are still reluctant to use telemedicine platforms due to at least six key factors, including lack of trust in such digital platforms, lack of accessibility, digital illiteracy, user-unfriendly platforms, lack of support, and inequalities related to gender, age, social groups, etc. Bakhtiar et al. (10) argue that if we do not concern about the risks of elevating disparities and providing less and poor-quality services to those most underserved patients, “*internet access and device ownership could become social determinants of health*.” There is an urgent need to foster and support equitable access to telemedicine. It is appreciated that Shen et al. (3) did not refer to telemedicine platforms as all-inclusive platforms. Still, we argue that telemedicine services are not yet equal to traditional care systems. There are still significant barriers, including but not limited to the digital divide barriers, which need to be addressed. Other barriers include a lack of standardized telemedicine pathways and poor digital literacy (11). Hence, we urge ongoing and future research to not neglect the presence and growing impacts of digital divide barriers in online or internet services. We have to appreciate the fact that certain groups have tangible barriers, and they should be addressed more promptly to optimize current (and future) telemedicine platforms and online healthcare services.

More specifically, the mental digital divide barrier (DDB) is the most intangible barrier, relying on people's willingness and intention to use telemedicine. Regarding the Unified Theory of Acceptance in Technology (UTAT), it is suggested that the most significant influential factors of an individual's intention to use telemedicine are performance expectations, effort expectations, and facilitating conditions (12). Facilitating conditions reflect the material DDB, while performance expectancy mainly depends on doctors' opinions, and the effort expectancy is closely linked to computer anxiety (12, 13). This implies that improving people's awareness and fostering behaviors change through political incentives and propaganda activities to boost individuals' perceptions and intentions to use telemedicine might be a possible solution to intangible mental DDBs.

Secondly, for material DDB of telemedicine, most telemedicine platforms require smartphone use. However, some people cannot even afford or get access, like the poor, the elders, and children. This situation might be mitigated or partially solved by sharing economy like providing public laptops, desktops, and smart devices in public places (e.g., streets, community activity centers, pharmacies, etc.). After overcoming the second DDB of material accessibility, education level, digital literacy, and learning abilities form the third DDB: skill DDB. Since digital advances and technologies develop rapidly, people with limited and/or less ability to adapt to rapid digital innovations and upgrades will all suffer from skill DDB. Not only are patients from the socially marginalized and/or disadvantaged groups impacted by skill DDB the most, but doctors and/or medical practitioners need to learn and adapt to the use of telemedicine systems to provide more accurate and effective diagnoses since they will be using them the most. On the other hand, usage of DDB is highly connected to mental and accessibility DDBs, behavioral intentions, related popularization & promotion of telemedicine platforms, and the fifth DDB: utility DDB.

Beyond mental, material, skill, and usage DDBs, we argue that there is another type of DDB interconnecting with all the four DDBs, utility DDB. The underlying factors of utility DDB can be divided into users' and providers' factors while they are closely intertwined with each other DDBs. For instance, some hospitals don't provide enough financial and technological support to build their telemedicine systems, making it very hard to use or useless to some extent. Consequently, this will deepen mental issues concerning the usage of DDBs while making digital literacy less relevant while such technologies may not be readily used by consumers due to cost and utility barriers. To resolve this, it is suggested that collaborations between academics and practitioners are required to develop the feasibility and utility of telemedicine in resource-limited settings. At the same time, emerging technologies like Artificial Intelligence and wearable devices are expected to mitigate DDBs of telemedicine and resolve their limitations (14).

Generally, those neglected barriers can be mitigated through an integrated approach from macro, meso, and micro levels. At macro level, strategies include implementing national elder-orientated standards and regulations for telemedicine systems/platforms (e.g., elder's mode, simple mode, in-app training sessions, AI, and other supportive features). They promote a systematic transformation of medicine systems to minimize health system–created barriers [e.g., provide incentives for hospitals to build better telemedicine systems through top-down approaches, advocate for policies, and infrastructure that facilitate equitable telemedicine access (15). They also integrate scopes and functions of social welfare institutions for more inclusive service objects (i.e., not only the disabled groups but also other vulnerable population groups); and smart and dynamic management protocols with a particular classification of people with special needs. At meso levels, local government and communities can play very significant roles in mitigating the DDB of telemedicine. Not all the DDBs can be resolved completely, particularly for skill and utility DDBs, but these strategies can be applied. First, by setting special channels at the hospital to enhance inclusivity; and target developing digital vulnerable people-friendly cities/communities, e.g., age-friendly communities (16, 17). Second, by setting public desktops booth on the streets/parks or combining public libraries with Internet cafes]. This approach can create new jobs for assisting and supporting those digital vulnerable people to get the most benefits of telemedicine (e.g., home telemedicine consultants, social workers, or volunteers). Thirdly, an individual's skill and mental DDBs can be dealt with more effectively and precisely at micro-levels. Such mitigation strategies may involve awareness enhancement/training programs, and more user-friendly interfaces. Some examples include in-app training modes, AI assistant robots (18), chatbots (19), etc. (20). Lastly, we could set up programs and courses to train medical practitioners to use telemedicine by offering related courses at universities for seniors.

## Author Contributions

All authors listed have made a substantial, direct, and intellectual contribution to the work and approved it for publication.

## Funding

AC acknowledges the National Natural Science Foundation of China (NSFC) for the provision of funding for project number 71950410760. He also acknowledges the Ministry of Education, Culture, Sports, Science, and Technology (MEXT), Japan Government, and the Network for Education and Research on Peace and Sustainability (NERPS), Hiroshima, Japan.

## Conflict of Interest

The authors declare that the research was conducted in the absence of any commercial or financial relationships that could be construed as a potential conflict of interest.

## Publisher's Note

All claims expressed in this article are solely those of the authors and do not necessarily represent those of their affiliated organizations, or those of the publisher, the editors and the reviewers. Any product that may be evaluated in this article, or claim that may be made by its manufacturer, is not guaranteed or endorsed by the publisher.
